# Behavioural and electrophysiological responses of *Philaenus spumarius* to odours from conspecifics

**DOI:** 10.1038/s41598-022-11885-3

**Published:** 2022-05-19

**Authors:** Milos Sevarika, Gabriele Rondoni, Sonia Ganassi, Onofrio Marco Pistillo, Giacinto Salvatore Germinara, Antonio De Cristofaro, Roberto Romani, Eric Conti

**Affiliations:** 1 Department of Agricultural, Food and Environmental Sciences, University of Perugia, 06121 Perugia, Italy; 2 Department of Agricultural, Environmental and Food Sciences, University of Molise, 86100 Campobasso, Italy; 3 Department of Agriculture, Food, Natural Resources and Engineering, University of Foggia, 71122 Foggia, Italy

**Keywords:** Behavioural ecology, Invasive species, Animal behaviour

## Abstract

The meadow spittlebug, *Philaenus spumarius* L. (Hemiptera: Auchenorrhyncha: Aphrophoridae), is the main vector of *Xylella fastidiosa* subsp. *pauca* strain ST53, the causal agent of the Olive Quick Decline Syndrome. *Philaenus spumarius* and other Auchenorrhyncha are known to communicate via vibrations, whereas the possible occurrence of semiochemical communication has been poorly investigated so far. Through a chemical ecology approach, we provide evidence of intraspecific chemical communication in *P. spumarius*. In Y-tube olfactometer bioassays, males were attracted to unmated females as well as toward the headspace volatile extracts collected from unmated females. Conversely, females did not respond to unmated male volatiles or their extracts, nor did males and females respond to volatiles from individuals of the same sex. Electroantennography assays of unmated male and female headspace extracts elicited measurable responses in the antennae of both sexes. Male responses to body wash extracts from both sexes were stronger compared to female responses. Thus, suggesting the presence of compounds that are highly detected by the male’s olfactory system. The female head seemed to be the source of such compounds. This is the first record of intraspecific chemical communication in *P. spumarius* and one of the very few records in Auchenorrhyncha. Possible biological roles are under investigation.

## Introduction

The meadow spittlebug, *Philaenus spumarius* L. (Hemiptera: Aphrophoridae), is an important vector of the invasive gamma-proteobacterium *Xylella fastidiosa* Wells et al. subsp. *pauca*, strain ST53, which is the causal agent of the Olive Quick Decline Syndrome (OQDS) in Italy^1^. This xylem-limited obligate plant pathogen is transmitted between plants by xylem sap-feeding insects belonging to different families of Cicadomorpha (Hemiptera: Auchenorrhyncha) and causes significant losses to several crops^2,3^. Native to the Americas, *X. fastidiosa* was accidentally dispersed in Asia (Iran, Israel, Taiwan) and Europe (France, Spain, Portugal, Italy)^3–6^. The ST53 strain of *X. fastidiosa* subsp. *pauca* was recorded in southern Italy by Saponari et al.^1^ and is presently spreading across the Apulia region. Several studies were conducted to identify the native vectors of this accidentally introduced bacterium. In addition to *P. spumarius,* which appears to play a major role in the olive growing areas, other identified vectors are *Neophilaenus campestris* Fallen and *Philaenus italosignus* Drosopoulos et Remane (Hemiptera: Aphrophoridae)^1,7–9^.

To date no successful management strategies have been developed against *P. spumarius*, mostly because this species was not a pest before the arrival of *X. fastidiosa* in Italy. The number of biocontrol agents is relatively low, and they do not provide satisfactory reduction of *P. spumarius* populations^10^. Currently, mandatory actions taken to control vector populations are weeding or mowing in olive grove areas to reduce *P. spumarius* nymph populations and application of pyrethroids (e.g., deltamethrin) and neonicotinoids on olive plants against the adults with well recognized risks of side effects on natural enemies^11,12^.

*Philaenus spumarius* and Auchenorrhyncha in general are known to communicate via substrate-borne vibrations^13^. Specifically, Avosani et al.^14^ have identified six distinct vibrational signals used by *P. spumarius*. Use of vibrations as a way to control a pest was shown to be effective in the case of the leafhopper *Scaphoideus titanus* Ball (Hemiptera: Cicadellidae)^15^. However, application of this method to *P. spumarius* needs to be tested. On the other hand, knowledge on the involvement of semiochemicals in communication by Auchenorrhyncha is very poor, although this has been intensively investigated in other Hemiptera, such as Sternorrhyncha, especially Aphididae^16^, Psyllidae^17^, Pseudococcidae^18^, and Heteroptera^19–21^.

Morphological and ultrastructural investigations apparently seem to confirm the lower importance of olfactory cues for Auchenorrhyncha. *Philaenus spumarius* antennae have significantly fewer antennal sensilla compared to other species^22^. Nonetheless, electrophysiological assays showed that *P. spumarius* adults respond to 50 different volatile organic compounds belonging to six different groups^23^, and behavioural observations showed that different essential oils were attractive or repellent^24^. Very little research has been published on pheromones in Auchenorrhyncha. The presence of alarm pheromones has been investigated in adults and nymphs of Membracidae^25^, and an aggregation pheromone was identified in nymphs of the rice spittlebug *Callitettix versicolor* (Fabricius) (Hemiptera: Cercopidae)^26^. Based on a morphological study, Liang^27^ hypothesized the presence of glands on the head of adult *C. versicolor* males, that may produce a pheromone, with a possible role as short range attractant or aphrodisiac.

We hypothesized that for intraspecific communication, in addition to vibrational cues that are involved in sexual communication from a short distance on the plant substrate^28,29^, *P. spumarius* might have developed the ability to exploit volatile chemical cues, possibly acting from long distance. This role could possibly be played by plant volatiles, as it happens with *Anastrepha fraterculus* (Wiedemann) (Diptera: Tephritidae)^30^ and partially with *Lygus rugulipennis* Poppius (Hemiptera: Miridae)^31,32^, and/or by pheromones, as shown in several Hemiptera species^33,34^. Here we tested the possible involvement of chemical stimuli in intraspecific communication of unmated *P. spumarius* adults through behavioural and electroantennographic (EAG) investigations. In Y-tube olfactometer bioassays, we evaluated the behavioural responses of males and females of *P. spumarius* to volatiles emitted by adults of both sexes and to their headspace extracts. Additionally, we tested the EAG responses of *P. spumarius* males and females to headspace and body wash extracts.

## Results

### Olfactometer bioassays

**Figure 1:**
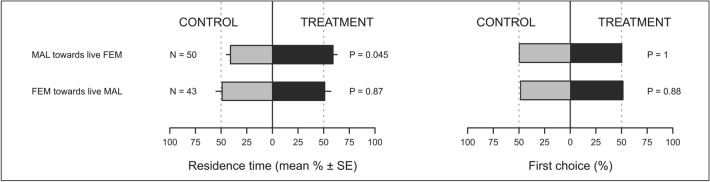
Residence time (mean % ± SE) and first choice (%) of *Philaenus spumarius* males (MAL) and females (FEM) in the control and treatment arm of a Y-tube olfactometer. Control consisted of clean air. Treatments were volatiles from live *P. spumarius* females or males. Data were analysed by means of GLS (residence time) or GLM with binomial distribution (first choice).

**Figure 2:**
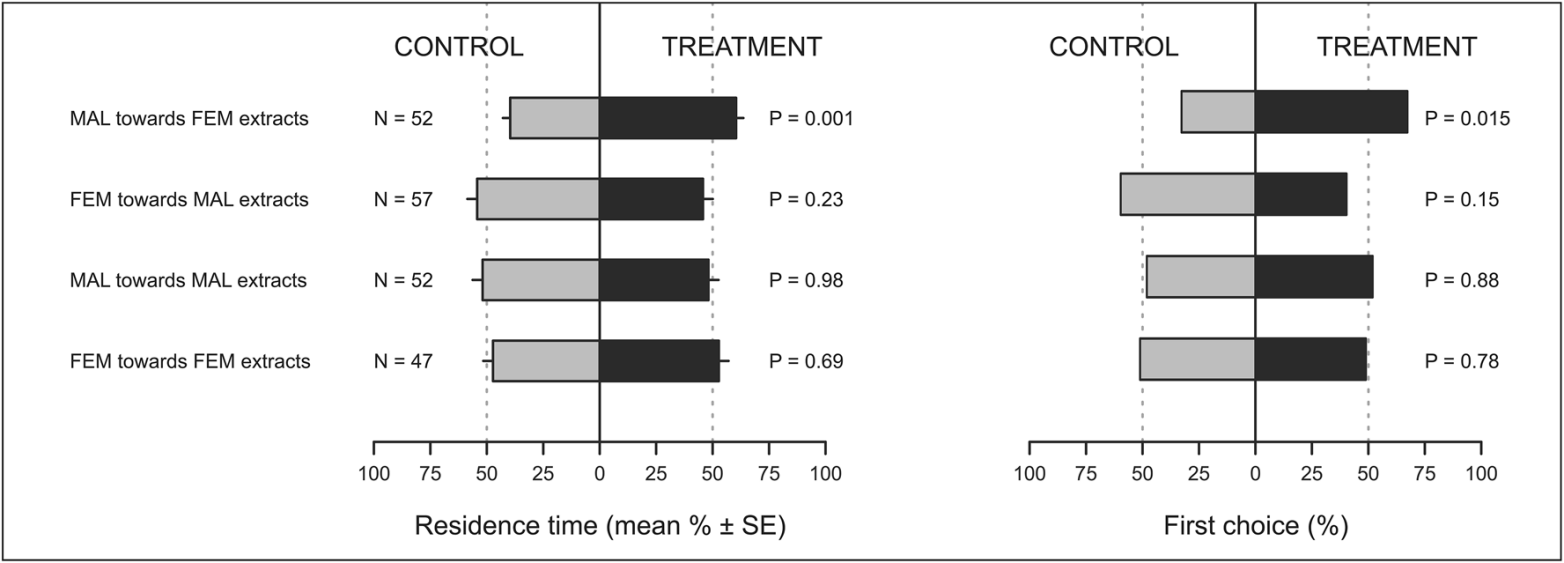
Residence time (mean % ± SE) and first choice (%) of *Philaenus spumarius* males (MAL) and females (FEM) in the control and treatment arm of a Y-tube olfactometer. Control consisted in dichloromethane. Treatments were headspace volatiles collected from live *P. spumarius* females or males using dichloromethane as solvent. Data were analysed by means of GLS (residence time) or GLM with binomial distribution (first choice).

When exposed to odours from two live insects (Fig. 1), *P. spumarius* males spent a greater proportion of their time in the olfactometer arm containing volatiles from live females compared to the control (GLS, t-value = 2.03, df = 91, P = 0.045). Conversely, *P. spumarius* females did not respond to odours from unmated males (t-value = 0.16, df = 91, P = 0.87). No behavioural differences were detected in the first choices of males or females toward treatments vs. controls (P > 0.05 for both comparisons). When exposed to extracts of headspace volatiles collected from four live conspecifics (Fig. 2), males positively responded to extracts from females showing higher residence time in the treatment arm compared with control arm (t-value = 3.32, df = 204, P = 0.001), whereas females did not respond to male extracts (t-value = − 1.19, df = 204, P = 0.23). Neither males or females responded to extracts from conspecifics of the same sex (males vs. males: t-value = − 0.02, df = 204, P = 0.98; females vs. females: t-value = 0.39, df = 204, P = 0.69). Data on males first choices confirmed their positive response to female extracts (binomial GLM, χ^2^ = 6.0, df = 1, P = 0.015), whereas all the other treatments were not different from controls (P > 0.05).

### EAG responses

**Figure 3:**
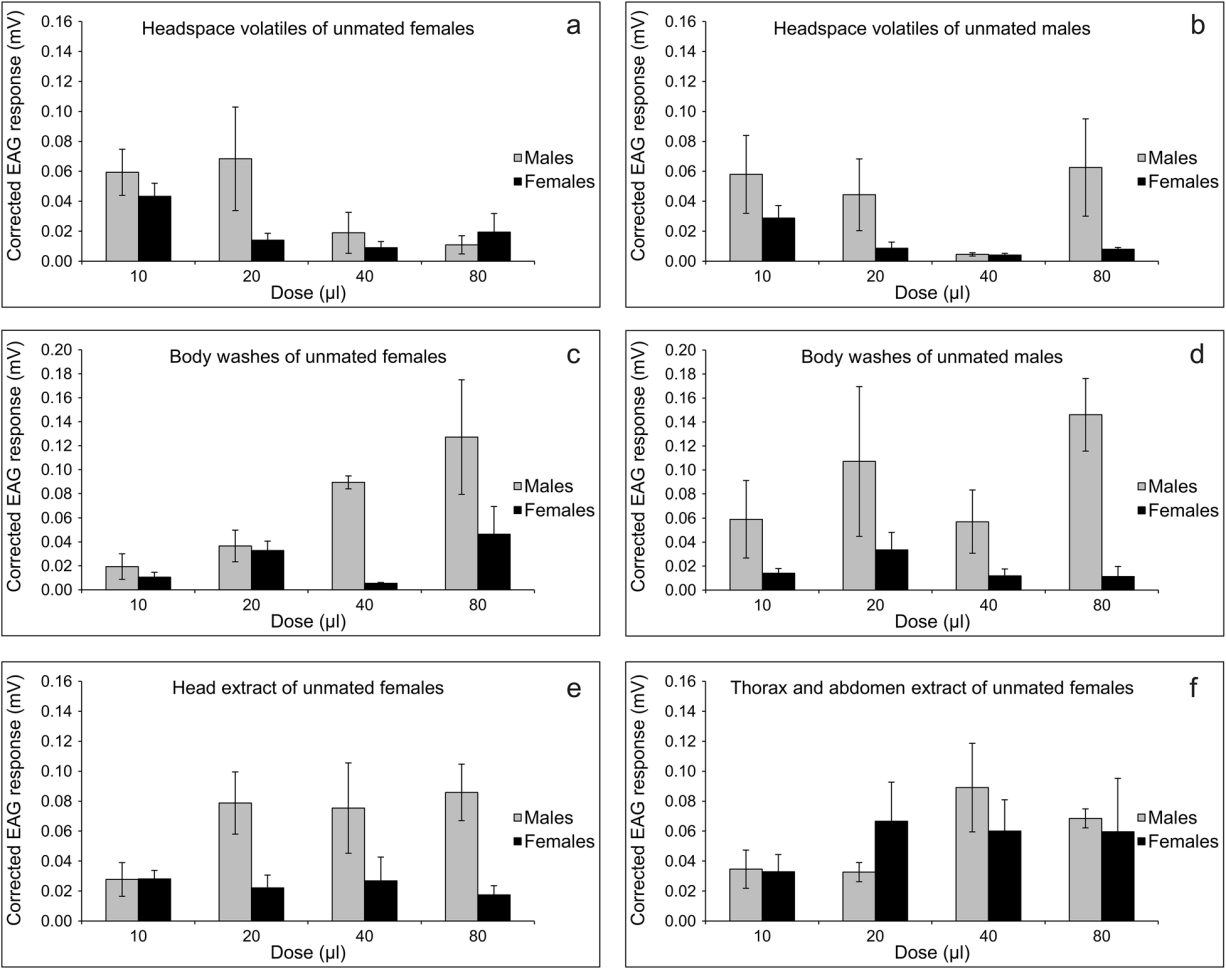
Electroantennography (EAG) responses (mean mV ± SE) of unmated *P. spumarius* males (grey bars) and females (black bars) to different doses of the stimuli. Stimuli consisted of headspace extracts of 15 unmated females (**a**) or males (**b**), or of body wash extracts of 8 unmated females (**c**) or males (**d**), or of extracts of head (**e**) or thorax and abdomen (**f**) of unmated females. Male and female mean EAG responses at the doses tested were compared by LMM for repeated measures, eventually followed by multiple comparisons procedure. Sex × dose interaction was never significant. Body washes of unmated females or males and head extract of unmated females elicited higher EAG response in males compared to females. Additionally, there was a positive dose–response effect to body washes of unmated females, with lower response at 10 μL compared to response at 80 μL.

Concerning EAG responses (Fig. 3), the activation threshold was always 10 μL for females (one-sided t-tests, females: t ≥ 2.54, df = 4, P ≤ 0.032) or males (t ≥ 2.23, df = 4, P ≤ 0.045), with the exception of 20 μL for males exposed to body washes of unmated females (t = 2.77, df = 4, P = 0.025).

EAG responses for sex were always independent of doses for the different extracts (F_3,24_ ≤ 2.37, P ≥ 0.10; Tables S1–S6). Male antennal response was greater than that of females to body washes of unmated females (F_1,8_ = 9.68, P = 0.014, Table S3), body washes of unmated males (F_1,8_ = 13.91, P = 0.006, Table S4), or head extract of unmated females (F_1,8_ = 13.54, P = 0.006, Table S5). Additionally, there was a significant effect of dose on antennal response to body washes of unmated females (F_3,24_ = 4.56, P = 0.001), with a positive dose–response trend (10 μL vs. 80 μL: P = 0.011; P > 0.05 for all the other comparisons, Table S3). There was no effect of dose on antennal response for all the other extracts (F_3,24_ ≤ 2.97, P ≥ 0.052, Tables S1, S2, S4–S6).

## Discussion

Hemipterans encompass many species characterized by multimodal communication. Use of vibration and sex pheromones in intraspecific communication is known for several superfamilies of Heteroptera (Pentatomoidea, Miroidea, Reduvioidea, Cimicoidea, Gerroidea) and Sternorrhyncha (Aphidoidea, Psylloidea and Aleyrodoidea)^13,16,17,19,21,35,36^. However, with very few exceptions, namely an aggregation pheromone in juveniles of a species of Cercopidae^26^ and alarm pheromones in juveniles and adults of some Membracidae^25^, almost nothing is known on pheromonal communication in the case of Auchenorrhyncha, which instead are known to exploit vibrational cues^13^. Here, using unmated males and females of *P. spumarius*, we provide for the first time in adult Auchenorrhyncha, evidence of intraspecific communication involving attraction to volatiles from conspecifics.

In olfactometer bioassays, *P. spumarius* males responded positively to unmated live females and to volatiles released by unmated females, whereas they did not respond to volatiles from males, indicating ability to discriminate between male and female chemical profiles. Unmated females did not exhibit any response to unmated males or to volatiles emitted by both sexes. Although these results might suggest a possible role in sexual communication of female-emitted volatile compounds, additional investigations are necessary before any hypothesis can be proposed. The male responses were stronger when using headspace volatile extracts rather than live females. This is not surprising because the volatile emission by females might not be regular in time and there is no guarantee that any active compound is emitted during the 10-min duration of the single behavioural observation. In contrast, the headspace volatiles were collected for a period of 3 h, which increased the probability that behaviourally active chemicals were collected in the extracts, and then evenly dispensed during the different bioassays.

In EAG recordings, the headspace volatile extract and body washes of males and females of *P. spumarius* elicited antennal responses in both sexes, thus demonstrating the capability of the olfactory receptors to detect volatile compounds present in the extracts. When the headspace volatiles were tested, electrophysiological responses did not differ between sexes. However, when the body wash extract from unmated females was assayed, EAG responses were significantly higher in males than females. The body wash method allows collection of compounds both internally and externally. Therefore, a complete profile can be obtained, including possible contents of insect glands that are not emitted during headspace extraction. Similar results were observed in the case of *Diaphorina citri* Kuwayama (Hemiptera: Liviidae)*,* in which airborne volatiles did not induce response of males toward female volatiles in Y-tube olfactometer, while direct solvent extracts from females induced significant male responses^37^. Surprisingly, when the body wash of unmated males of *P. spumarius* was assayed, EAG responses were higher in males compared with females. While the higher response of male antennae to female compounds seems consistent with possible existence of a sex attractant in *P. spumarius*, the reason for the strong response of male antennae to male body wash is unknown, confirming the need of further investigations. Remarkably, when washes from the female head were tested, the EAG response of male antennae was higher compared with female response, whereas when washes from the female thorax and abdomen were assayed, male and female responses were not significantly different. This suggests that the source of volatile compounds eliciting electrophysiological response in males could be located in the female head. Indeed, recent ultrastructural and morpho-functional investigations carried out on *P. spumarius* showed the presence of two morphologically different types of glands*.* Particularly glands type II, present in both sexes and located solely at the apical part of the postclypeus were proposed as potential pheromonal glands^38^.

The actual role of the female-emitted volatiles is under investigation. Like many Hemiptera, *P. spumarius* is known to use vibrational cues in sexual communication^14^. However, these vibrational cues likely act on a short distance, whereas chemical cues may act on a longer distance. Therefore, combined exploitation of vibrational and chemical cues by *P. spumarius* in intraspecific and even sexual communication cannot be excluded, also considering that most insects have adapted to use multimodal communication and these include many Hemiptera^20,33,39^. Additionally, we must consider that in our study we used 2- to 3-week-old individuals that were field collected in the nymphal stage and then separated in the adult stage to obtain unmated males and females. This procedure is necessary when starting investigation on sex pheromones. However, it is possible that females could become receptive to volatiles later, for example after egg maturation. *Philaenus spumarius* seems to mate late in the season, when individuals are older, as also deduced from the knowledge that females of *P. spumarius*, differently to males, are not able to produce vibrational calls soon after eclosion. Indeed their calling activity is correlated with ovariole development and the presence of mature eggs, which occur from July–August onwards, whereas before becoming sexually mature females only produce rejection vibrations in response to the male calling signals^14,40^. This information might also explain why in our investigation the amount of emitted compounds was quite low and it was necessary to concentrate the extracts to obtain electrophysiological responses. Future research should verify volatile emission and behavioural response in female *P. spumarius* when they are sexually mature and ready to mate. Whether male attraction towards female is consistent among different *P. spumarius* populations is also unknown. This species is highly polymorphic and colour patterns are under genetic control, whose distribution is known to be determined also by geographic constraints^41,42^. This aspect has been investigated in other systems. For instance, the very well-studied *Sesamia nonagroides* Lefèbvre (Lepidoptera: Noctuidae) has a fragmented distribution and genetic variability among populations^43^. Intriguingly, some geographically separated populations of this species exhibit differential expression of chemosensory genes involved in olfaction, possibly reflecting different adaptations to different environmental conditions, e.g., host plants^44^.

The identification of chemical volatiles that are attractive to *P. spumarius* adults would provide interesting new perspectives for management of this important vector of the invasive bacterium *X. fastidiosa*. Because of their migratory behaviour, *P. spumarius* adults at beginning of summer move from herbaceous plants to olive plants and other trees, while at the end of summer they move back to herbaceous plants^45^. Previous studies demonstrated that essential oils from different plant species can be attractive or repellent to adults of *P. spumarius* and were proposed for use in push–pull strategies^24,46^. Therefore, possible identification of active compounds can act as attractant(s) that, combined with repellents, might be considered in the future for application in *P. spumarius* control. Identification of volatile profiles of *P. spumarius* could also provide potential for its integration with biological control. Recently, it was shown that *P. spumarius* is parasitized by *Verrallia aucta* Fallén (Diptera: Pipunculidae) with the maximum parasitization rate of 17.5%^47^. In addition, this spittlebug was found to be variably parasitized (average parasitism ranging from 20 to 69%) in Corsica by *Ooctonus vulgatus* Haliday (Hymenoptera: Mymaridae), an egg parasitoid species that is present in most of Europe, including southern Italy^48^. Mymarid egg parasitoids exploit physical and chemical cues from the host and from the plant during host location^49–51^. Hence, once identified, volatile compounds from *P. spumarius* can be evaluated for potential use to increase the efficacy of egg parasitoids, as was proposed for other Hemiptera pests^52,53^. The integration of novel chemical ecology methods, which aim at keeping the *P. spumarius* populations out from the olive groves and in the meantime improving natural enemy efficacy on wild host plants, could be a promising and sustainable strategy to manage this important vector of *X. fastidiosa*.

In conclusion, we have provided evidence of intraspecific communication of *P. spumarius* mediated by volatile compounds. Further investigations are ongoing and aim to characterize the volatile profiles from both sexes and to identify chemical active compounds.

## Materials and methods

### Insects

Nymphs of *P. spumarius* were collected in April and May 2019 and 2020 from meadows in the Perugia (coordinates: 43.09154, 12.38907) and Colfiorito areas (coordinates: 43.03349, 12.90824) (Umbria, central Italy). Plants (e.g., *Bellis perennis* (L), *Dipsacus fullonum* (L), *Sonchus oleraceus* L, *Sorghum halepense* (L.), *Triticum aestivum* (L), and *Vicia faba minor* L.) bearing *P. spumarius* nymphs, indicated by the presence of the nymph-produced foam masses, were cut and transferred using plastic containers (30 cm × 20 cm × 10 cm) to the rearing facility. Using a fine brush, nymphs were gently transferred to *V. faba minor* (var. “Scuro di Torre Lama”, provided by Agroservice S.p.a., San Severino Marche, Macerata, Italy and certified by CREA-DC, Milano, Italy) plants and reared in mesh cages (Kweekkooi 40 cm × 40 cm × 60 cm, Vermandel, Hulst, The Netherlands) under controlled conditions (25 ± 1 °C, 60 ± 5% relative humidity, LD 16:8 h photoperiod). Nymphs were checked every two days for adult emergence. Freshly emerged adults, still unmated, were sexed under a stereomicroscope and separated by sex to prevent mating. Fresh potted *V. faba* plants were provided to insects every 10 days. For the experiments, 7- to 14-d old unmated males or females were used. The plant materials collected and used in the study and the research conducted comply with relevant institutional, national and international guidelines and legislation.

### Collection of headspace volatiles from insects

Headspace volatiles were collected from 4 or 15 unmated *P. spumarius* males or females. Extracts from 4 individuals were used for olfactometer bioassays. Extracts from 15 individuals were used for EAG recordings because during preliminary experiments the extracts from 4 individuals did not elicit significant responses. Volatiles were collected by pumping environmental air (diaphragm pump model NMP 830 KNDC 12 V, KNF Italia S.r.l., Milan, Italy) at 2.2 L/min through a flowmeter (Dwyer, U.K.) and then an activated charcoal filter (260 mm length, 40 mm internal diameter). Then, the air flow was split into two identical routes, each consisting of a Dreschel bottle (250 mL volume) containing unmated *P. spumarius* males or females. A disk of filter paper (5 cm diameter) was included in the bottle and moistened with 1 mL of tap water. The Dreschel bottle consisted of a glass jar topped with a glass lid provided with an inlet tube, reaching the bottom of the jar, and a short outlet. The outlet was connected to a glass tube (8 cm length, 6 mm internal diameter) filled with 70 mg of Porapak Q (80–100 mesh size, Alltech, Sedriano, Italy) which allowed trapping of volatile compounds. The air was pumped out from the trap at 900 mL/min by an additional diaphragm pump positioned at the end of the device. Teflon tubes (3 mm internal diameter) conveyed the airflow through the system and were connected to the different parts using silicone seals and brass connectors. Drilled Teflon cones (6 mm internal diameter) and brass connectors were used to connect tubes with glass Porapak Q traps. Parafilm was then used to seal all the connections.

Volatiles were collected for 3 h from unmated adult individuals, from 09:00 to 12:00. Because starvation may cause insect mortality (usually after 4 h, personal observation), prior to volatile collection insects were maintained on host plants.

Volatiles were eluted from Porapak Q in glass vials with 700 µL of dichloromethane (99%, VWR chemicals, Fontenay-sous-Bois, France). Elutions were successively concentrated to approximately 350 µL under a gentle nitrogen stream. Vials were closed with Teflon cap liners and kept at −20 °C until extracts were evaluated in bioassays. Before use, Dreschel bottles, glass vials and taps were washed with laboratory detergent and rinsed with tap water and dichloromethane and kept overnight at 110 °C. Porapak Q traps were washed 4–5 times with 500 mL dichloromethane. Then, Porapak Q traps, teflon tubes and charcoal filters were conditioned overnight at 110 °C under a nitrogen stream.

Volatile collection was conducted in an environmental-controlled room (24 ± 1 °C, 60 ± 5% RH) under four fluorescent lamps (Philips Master TLD 58 W/840). Those replications where one or more insects were dead at the end of the 3-h volatile collection were discarded. Five to 10 replications per treatment were successfully conducted.

### Olfactometer bioassays

The effect of volatile treatments on the behaviour of unmated male and female *P. spumarius* was investigated in a Y-tube olfactometer (stem 90 mm; arms 80 mm angled 130 degrees, internal Sect. 15 mm × 10 mm), carved in polycarbonate plate (200 mm × 190 mm × 10 mm thick) sandwiched between two glass sheaths (each plate: 200 mm × 150 mm × 5 mm thick), providing the upper and lower closure of the olfactometer, respectively. Air flowed from a tank of medical-grade compressed air (N2/O2 80:20) and was diverged in two identical routes. Each route consisted of a flow meter, that regulated the air flow at 0.25 L/min, a water jar to humidify the air and a glass chamber containing the volatile source (e.g., live insects or headspace volatile extract). The glass chamber consisted of a modified Dreschel bottle (250 mL volume), where the cylindrical bottle flask was replaced by a spherical glass flask of the same volume. The air was then conveyed in one of the two arms of the olfactometer. Medical silicone tubes (6 mm internal diameter) were used to connect all parts of the device (full schematics of the olfactometer device is reported in^54^, supplementary material). The system was surrounded by a black fabric curtain to minimize possible visual cues from the room that could affect insect response. The device was illuminated from above by two 22-W cool white fluorescent tubes and from below by infrared lights (homogeneous emission of wavelengths at 950 nm, provided by 108 LEDs). Bioassays were conducted from 09:00 to 14:00 in a temperature-controlled room at 25 °C.

Two experiments were performed to evaluate the effect of two types of odour sources (live insects or headspace extracts of insect volatiles) on *P. spumarius* behaviour. In the first experiment, we tested whether *P. spumarius* males were attracted toward live females or whether females were attracted toward live males, compared to control (clean air) as follows. Two *P. spumarius* females or males were placed in the glass flask. A filter paper (5 cm diameter) wetted with 0.5 mL of tap water and a piece of brass mesh (6 cm length, 2 cm wide) were included in the chambers. The brass mesh provided the insects with a surface they could grasp with their tarsi, and allowed them to right themselves if they fell over on their backs. The control chamber included only wetted filter paper and a clean piece of brass mesh. We conducted 50 replicates with individual males in response to females and 43 replicates with individual females in response to males.

For the bioassays, a single adult of *P. spumarius* was gently introduced into the central stem of the Y-tube. Insects’ behaviour was recorded continuously for 10 min with a monochrome CCD video camera (Sony SSC M370 CE) fitted with a 12.5–75 mm/F1.8 zoom lens, covered with an infrared pass filter (Kodak Wratten filter 87A). Analog video signal coming from the camera was digitized by a video frame grabber. Data were processed by Xbug, a video tracking and motion analysis system^55^.

In the second experiment, we tested whether *P. spumarius* males or females were attracted toward headspace volatiles. We conducted four bioassays: (1) response of males to headspace volatiles collected from females; (2) response of females to volatiles collected from males; (3) response of males to volatiles collected from males; and (4) response of females to volatiles collected from females. The treatment (50 µL of a given extract) or control (50 µL of dichloromethane) was applied to a piece of filter paper (5 cm diameter) which was inserted inside the glass chamber. Forty-seven to 57 replicates were conducted per each bioassay.

After a series of five bioassays, the tubes entering to Y-tube arms were switched, so to avoid possible localization bias. At the same time, the olfactometer and glass plates were replaced with a clean set. Plastic parts were washed with laboratory neutral detergent, rinsed with tap water and air-dried. Glass parts were washed with detergent and acetone (99%, VWR chemicals, Fontenay-sous-Bois, France). Glasses were then dried in the oven at 110 °C for few hours. After 10 tested individuals, treatments were replaced with a new group of individuals or a new extract. In total, 10 individuals were tested with one extract/group of individuals.

### Body wash

Wash extracts were obtained from intact *P. spumarius* males and females, as well as from heads or thorax plus abdomen. Unmated *P. spumarius* individuals were anaesthetized for 1 min at −18 °C. Insect regions were eventually separated under a stereo microscope by micro scissors. Then, two groups of four individuals/body regions each were placed in two 1.5 mL glass vials, each filled with 500 µL of dichloromethane. Intact insects or body parts were kept submerged for 2 min, then the solvent of the two vials was collected using a syringe (Hamilton, USA) and blended in a new 1.5 mL vial to reach the total volume of 1000 µL. For each extract type, three 1000 µl samples were collected from each sex, i.e., 24 males and 24 females were sampled in total.

### Electroantennography (EAG) recordings

The EAG responses of *P. spumarius* male and female antennae to increasing amounts (10, 20, 40, 80 μL) of each stimulus (volatiles collected from 15 unmated females or 15 unmated males; body wash of 8 unmated females or 8 unmated males) were measured by the EAG technique similar to that used in previous studies^23,56^. For the test, a single adult was dissected between the head and the thorax and a glass micropipette (0.2–0.3 mm internal diameter) filled with 0.1 M KCl solution, acting as the indifferent electrode, was inserted into the head. The last antennal segment was put in contact with the end of a similar pipette which provided the recording electrode. Silver chloride wires were used to maintain the electrical continuity between the antennal preparation and an AC/DC UN-6 amplifier in DC mode connected to a PC equipped with the EAG 2.0 software (Syntech, Kirchzarten, Germany). A stream of charcoal-filtered humidified air (500 mL/min) was directed constantly onto the antenna through a stainless-steel delivery tube (1 cm i.d.) with the outlet positioned at approximately 1 cm from the antenna. Each test stimulus was absorbed onto a filter paper (Whatman No. 1) strip (1 cm × 2 cm) inserted in a Pasteur pipette (15 cm long) and used as an odour cartridge. Over 1 s, 2.5 cm^3^ of vapour from an odour cartridge were blown by a disposable syringe into the air stream flowing over the antennal preparation. Intervals between stimuli were 1 min. The control (80 μL dichloromethane) and the standard (20 μL of (*Z*)-3-hexen-1-ol) stimulus were applied at the beginning and at the end of the experiment, and in addition, the standard stimulus was applied after each group of two test odours, to evaluate the gradual decrease in the antennal sensitivity over time. The extracts were stored at −20 °C until use. The EAG responses were recorded from five antennae of different males and females. The amplitude (mV) of the EAG response to each test stimulus was adjusted to compensate for solvent and/or mechanosensory artefacts according to Raguso & Light^57^.

### Data analysis

*Philaenus spumarius* behaviour was described by the residence time, i.e., the proportion of time spent by a male or a female in each of the two arms of the olfactometer over the total time spent in both treatment and control arms. Those insects that did not exhibit a choice or that visited either arm for a total time of less than 30 s were discarded from the analysis. Additionally, the first choice, i.e., the first entrance of each insect in either olfactometer arm, was recorded. Prior to statistical analysis, the residence time in the treatment arm was subjected to logit transformation. This procedure ensured that only one measurement per insect was included in the analysis^54^. For the two experiments (odours from live insects or from headspace extracts), the significance of the insect response to each treatment was evaluated using Generalised Least Squares models (GLS), to account for heteroscedasticity among treatments^58,59^. Generalized Linear Models (GLMs, logit link, Binomial error distribution) were fitted to test the first choice on the treatment vs. control arm. For both residence time and first choice data, the effect of blocks (each block was a replicate of headspace volatile source) was initially evaluated as a random effect in generalized mixed models, but its relevance was never justified according to likelihood ratio test (LRT)^60,61^.

In dose–response curves, the activation threshold was considered to be the first dose at which the mean response was higher than “0” value using Shapiro–Wilk test for normality followed by one-sample Student’s t-test (P = 0.05, one-sided)^23,62^. For EAG responses to the different volatile extracts, the effects of sex, dose, and sex × dose interaction were analysed using Linear Mixed Effects Models (LMMs) for repeated measures (within different doses). In case of a significant effect, multiple comparisons procedure with Sidak correction was eventually conducted. Statistical analyses were performed under R statistical environment^63^, libraries *nlme*^64^, *MASS*^65^, *ggplot2*^66^, and *emmeans*^67^.

## Supplementary Information

Supplementary Information.

## Data Availability

The datasets used and/or analysed during the current study available from the corresponding author on reasonable request.
