# Supplementary material for: Behavioural and electrophysiological responses of Philaenus spumarius to odours from conspecifics
Source: Sci Rep. 2022 May 19;12:8402. doi: 10.1038/s41598-022-11885-3 (PMC9120500; doi:10.1038/s41598-022-11885-3)
Supplement: Supplementary file 1 — Supplementary Information. [file 41598_2022_11885_MOESM1_ESM.docx]

**BEHAVIOURAL AND ELECTROPHYSIOLOGICAL RESPONSES OF *PHILAENUS SPUMARIUS* TO ODOURS FROM CONSPECIFICS**

Milos Sevarika^1a^, Gabriele Rondoni^1a^, Sonia Ganassi^2^, Onofrio Marco Pistillo^3^, Giacinto Salvatore Germinara^3^, Antonio De Cristofaro^2^, Roberto Romani^1b^, Eric Conti^1b^

^1^Department of Agricultural, Food and Environmental Sciences, University of Perugia, Perugia 06121, Italy

^2^Department of Agricultural, Environmental and Food Sciences, University of Molise, Campobasso, 86100, Italy

^3^Department of Agriculture, Food, Natural Resources and Engineering, University of Foggia, Foggia, 71122, Italy

^a^ Shared first authorship; ^b^ Shared senior authorship

Corresponding authors: Gabriele Rondoni; email: [gg.rondoni@gmail.com](mailto:gg.rondoni@gmail.com); Giacinto Salvatore Germinara; email: [giacinto.germinara@unifg.it](mailto:giacinto.germinara@unifg.it); Antonio De Cristofaro; email: [decrist@unimol.it](mailto:decrist@unimol.it)

**Supplementary information**

Table S1: Significance of the different terms retained in linear mixed effect model on the EAG responses of male and female *Philaenus spumarius* at the different doses of headspace volatiles of unmated females.

|  | numDF | denDF | F-value | P |
| --- | --- | --- | --- | --- |
| (Intercept) | 1 | 24 | 30.00 | <0.0001 |
| sex | 1 | 8 | 2.57 | 0.147 |
| dose | 3 | 24 | 2.97 | 0.052 |
| interaction sex:dose | 3 | 24 | 1.48 | 0.246 |

Table S2: Significance of the different terms retained in linear mixed effect model on the EAG responses of male and female *Philaenus spumarius* at the different doses of headspace volatiles of unmated males.

|  | numDF | denDF | F-value | P |
| --- | --- | --- | --- | --- |
| (Intercept) | 1 | 24 | 15.04 | 0.001 |
| sex | 1 | 8 | 4.51 | 0.067 |
| dose | 3 | 24 | 2.12 | 0.124 |
| interaction sex:dose | 3 | 24 | 0.95 | 0.433 |

Table S3: Significance of the different terms retained in linear mixed effect model on the EAG responses of male and female *Philaenus spumarius* at the different doses of body washes of unmated females. Results of comparisons for sex and dose levels are provided.

|  | | numDF | denDF | | F-value | | P |  |  |  |
| --- | --- | --- | --- | --- | --- | --- | --- | --- | --- | --- |
| (Intercept) | | 1 | 24 | | 42.13 | | <0.0001 |  |  |  |
| sex | | 1 | 8 | | 9.68 | | 0.014 |  |  |  |
| dose | | 3 | 24 | | 4.56 | | 0.012 |  |  |  |
| interaction sex:dose | | 3 | 24 | | 2.37 | | 0.096 |  |  |  |
|  |  | | |  | |  | | |  |  |
| Planned comparisons | Estimate | | | SE | | t-value | | | P |  |
| sex (M vs F) | 0.04 | | | 0.01 | | 3.11 | | | 0.014 |  |
| dose (10 vs 20) | -0.02 | | | 0.02 | | -0.99 | | | 0.759 |  |
| dose (10 vs 40) | -0.03 | | | 0.02 | | -1.63 | | | 0.383 |  |
| dose (10 vs 80) | -0.07 | | | 0.02 | | -3.58 | | | 0.008 |  |
| dose (20 vs 40) | -0.01 | | | 0.02 | | -0.64 | | | 0.917 |  |
| dose (20 vs 80) | -0.05 | | | 0.02 | | -2.59 | | | 0.071 |  |
| dose (40 vs 80) | -0.04 | | | 0.02 | | -1.95 | | | 0.234 |  |

Table S4: Significance of the different terms retained in linear mixed effect model on the EAG responses of male and female *Philaenus spumarius* at the different doses of body washes of unmated males. Result of comparison for sex levels is provided.

|  | | numDF | denDF | | F-value | | P |  |  |
| --- | --- | --- | --- | --- | --- | --- | --- | --- | --- |
| (Intercept) | | 1 | 24 | | 30.25 | | <0.0001 |  |  |
| sex | | 1 | 8 | | 13.91 | | 0.006 |  |  |
| dose | | 3 | 24 | | 1.30 | | 0.297 |  |  |
| interaction sex:dose | | 3 | 24 | | 1.12 | | 0.360 |  |  |
|  | |  |  | |  | |  |  |  |
| Planned comparison | Estimate | | | SE | | t-value | | | P |
| sex (M vs F) | 0.07 | | | 0.02 | | 3.73 | | | 0.006 |

Table S5: Significance of the different terms retained in linear mixed effect model on the EAG responses of male and female *Philaenus spumarius* at the different doses of head extract of unmated females. Result of comparison for sex levels is provided.

|  | | numDF | denDF | | F-value | | P |  |  |
| --- | --- | --- | --- | --- | --- | --- | --- | --- | --- |
| (Intercept) | | 1 | 24 | | 59.26 | | <0.0001 |  |  |
| sex | | 1 | 8 | | 13.54 | | 0.006 |  |  |
| dose | | 3 | 24 | | 0.96 | | 0.427 |  |  |
| interaction sex:dose | | 3 | 24 | | 1.65 | | 0.205 |  |  |
|  | |  |  | |  | |  |  |  |
| Planned comparison | Estimate | | | SE | | t-value | | | P |
| sex (M vs F) | 0.04 | | | 0.01 | | 3.68 | | | 0.006 |

Table S6: Significance of the different terms retained in linear mixed effect model on the EAG responses of male and female *Philaenus spumarius* at the different doses of thorax and abdomen extract of unmated females.

|  | numDF | denDF | F-value | P |
| --- | --- | --- | --- | --- |
| (Intercept) | 1 | 24 | 43.11 | <0.0001 |
| sex | 1 | 8 | 0.01 | 0.938 |
| dose | 3 | 24 | 1.51 | 0.238 |
| interaction sex:dose | 3 | 24 | 0.84 | 0.486 |
